# Impact of Thyroid Tissue Status on the Cut-Off Value of Lymph Node Fine-Needle Aspiration Thyroglobulin Measurements in Papillary Thyroid Cancer

**DOI:** 10.3389/bjbs.2021.10210

**Published:** 2022-01-12

**Authors:** L. Zhai, W. Jiang, Y. Zang, Y. Gao, D. Jiang, Q. Tian, C. Zhao

**Affiliations:** ^1^ Department of Abdominal Ultrasound, The Affiliated Hospital of Qingdao University, Qingdao, China; ^2^ Department of Ultrasound, Shandong Cancer Hospital and Institute, Shandong First Medical University and Shandong Academy of Medical Science, Jinan, China; ^3^ Health Management Center, The Affiliated Hospital of Qingdao University, Qingdao, China; ^4^ Department of Laboratory Medicine, The Affiliated Hospital of Qingdao University, Qingdao, China

**Keywords:** thyroid cancer, lymph node, fine needle aspiration, thyroglobulin, papillary

## Abstract

**Objective:** To study the optimal cut-off value of thyroglobulin measurement in a fine-needle aspiration (FNA-Tg) in diagnosing malignant lymph nodes and benign lymph nodes (LNs) according to the thyroid tissue status.

**Methods:** A total of 517 LNs were aspirated: 401 preoperative LNs, 42 LNs after subtotal thyroidectomy and 74 suspected LNs after total thyroidectomy. The cut-off value of FNA-Tg was obtained from receiver operating characteristic (ROC) analysis. The cut-off value with the best diagnostic performance was then obtained by comparing different cut-off values from other studies.

**Results:** LN FNA-Tg levels differed between preoperative and total thyroid disease (*p* < 0.001) and subtotal thyroidectomy and total thyroidectomy (*p* = 0.03), but not between preoperative and subtotal thyroidectomy (*p* = 1.00). Accordingly, those 443 LNs with preoperative and subtotal thyroidectomy were compared to those 74 without thyroid tissue. The optimal cut-off value in thyroid tissue group was 19.4 ng/ml and the area under the ROC curve (AUC) was 0.95 (95% CI 0.92–0.97). The optimal cut-off value in thyroid tissue absence group was 1.2 ng/ml and the AUC was 0.93 (0.85–0.98). After the analysis and comparison of multiple cut-off values, the optimal diagnostic performance was still found to be 19.4 ng/ml and 1.2 ng/ml.

**Conclusion:** The influential factors of FNA-Tg are still controversial, and the optimal cut-off value of FNA-Tg can be determined based on the presence or absence of thyroid tissue. FNA-Tg can be used as an important auxiliary method for diagnosing cervical metastatic LNs of thyroid cancer.

## Introduction

Differentiated thyroid cancer includes both papillary thyroid carcinoma (PTC) and follicular thyroid carcinoma (FTC), and accounts for ∼70% of all thyroid tissue malignancies (1, 2). The global incidence of thyroid cancer has been increased by 2.4–3 times in the last three decades, and this is mainly due to increased diagnostic rate of papillary subtypes (more than 80% of differentiated thyroid cancer) (3). Although the disease has a highly favorable prognosis, cervical lymph node (LN) metastasis is the main cause for the metastasis of PTC and the main factor for local recurrence (4, 5). Therefore, it is necessary to distinguish the metastatic lymph nodes (LNs) from benign reactive lymphadenitis, and accurate evaluation of LNs before surgery is crucial for selection of treatment strategies and surgical scope. High-resolution neck ultrasonography and ultrasound-guided fine needle aspiration cytology (FNAC) have been the standard procedures for diagnosing cervical metastatic LNs (6). However, the specificity of ultrasound remains low, and FNAC highly depends on the experience and ability of the operators and cytopathologist, especially for small LNs, necrosis, and cysts (7). Recently, thyroglobulin (Tg) measurement in the washout fluid of the needle used for FNAC aspiration (FNA-Tg) has proved a useful tool for improving the diagnostic accuracy of FNA (8–12), and also it has been recommended by the American Thyroid Association management guidelines in 2015 (6).

Thyroglobulin is produced exclusively by follicular cells, and so its expression in nonthyroidal tissue supports the diagnosis of LN metastasis and recurrence of PTC (13, 14). Many studies have shown that FNA-Tg has higher diagnostic sensitivity and specificity than FNAC. The combination of FNAC and FNA-Tg greatly improves the diagnostic efficiency of metastatic LNs (8–11). However, there are still some uncertainties with regard to the optimal cut-off value of FNA-Tg in preoperative and postoperative settings. Many studies have reported influencing factors of FNA-Tg, such as serum thyroglobulin, thyroglobulin antibody (TgAb) and thyroid stimulating hormone (TSH), but no consistent conclusion has been reached.

We broadly hypothesised that the status of thyroid tissue is the main influencing factor, and determined to define the optimal cut-off value of FNA-Tg according to the thyroid status and compare different cut-off values of FNA-Tg to obtain a cut-off value with the best diagnostic performance. The optimal cut-off value of FNA-Tg may can be determined based on the presence or absence of thyroid tissue and FNA-Tg may can be used as an important auxiliary method for diagnosing cervical metastatic LNs of thyroid cancer.

## Materials and Method

Between January 2017 and February 2019, a total of 517 patients (with 517 LNs) with suspicious LNs as detected by ultrasound in the Affiliated Hospital of Qingdao University were collected. The US features suggested metastatic LNs due to the characteristics of microcalcifications (diameter <2 mm), round shape (long diameter to short diameter ratio <2), absence of fatty hilum, cystic changes, calcifications, and peripheral vascularity (6). All the suspicious lymph nodes meet at least two of these characteristics. FNAC and FNA-Tg were measured in all patients and benign and malignant LNs were confirmed by histology. The Institution Review Board of our institution approved this retrospective study (QYFYWZLL 26057).

All patients underwent ultrasound examination with high-frequency linear-array transducers of 5 to 12 HZ. US-guided FNA was performed using a 22 gauge needle with a 5 ml syringe. The aspirate contents were smeared on slides, stained with Diff-Quik, reviewed for rapid on-site evaluation. The other smears are immediately immersed and fixed in 95% ethanol, and subsequently stained by hematoxylin and eosin in the pathology laboratory. The same residual needle aspirate was washed out in 1.0 ml normal saline for FNA-Tg measurement. The samples were immediately sent to the laboratory and stored at −22°C for 0–4 days for thyroglobulin analysis by an automated electrochemiluminescence immunoassay (Cobas e 801, Roche Diagnostics, Mannheim, Germany). The lower level of detection was 0.4 ng/ml, upper level was 500 ng/ml. The positive TgAb was measured with a reference value >115 μ/ml.

All FNAC results divided the LNs into positive and negative groups. The non-metastatic LN group included benign, inflammatory, atypical hyperplasia and inadequate cytology. The metastatic LN group included suspected malignant and malignant. The benign and malignant LNs were confirmed by surgical pathological results, which is the gold standard. All patients were tested for FNA-Tg, and only part of the patients measured serum thyroglobulin and TgAb levels.

SPSS 24.0 software was used for data analysis and image construction. Continuous variables were expressed as mean with standard deviation, and the classified variables were expressed as median (interquartile range). One-way analysis of variance (ANOVA) was performed for the normally distributed variables, non-parametric Kruskal-Wallis test was performed for the non-normally distributed variables. Receiver-operating characteristic analysis (ROC) was performed to determine the optimal diagnostic cutoff value of FNA-Tg in the diagnosis of LN metastasis under different thyroid tissue states. The area under the ROC curve (AUC) was obtained. The sensitivity, specificity, positive predictive value (PPV), negative predictive value (NPV) and diagnostic accuracy were used to compare the diagnostic value of the different diagnostic methods. Data with a *p* value < 0.05 was considered significant.

## Results

From 517 patients, 517 LNs were sampled (Table 1). There were 401 preoperative LNs, 42 LNs after subtotal thyroidectomy (combined into a group of 443) and 74 suspected LNs after total thyroidectomy. The baseline characteristic of lymph nodes are as shown in Table 1. Of the 517 samples, 198 were TgAb positive and 102 were surgical-positive. In those with LN metastasis, there was no significant difference in FNA-Tg between patients with positive TgAb and patients with negative TgAb (78.3 ng/ml [7.5–500.0] vs. 329.0 ng/ml [13.2–500.0], respectively; *p* = 0.84). FNA-Tg and serum thyroglobulin failed to correlate significantly in the thyroid tissue presence group (r = 0.01), with a weak positive correlation in the thyroid tissue absence group (r = 0.21). Seventy-four patients were treated with thyroxine-suppressive therapy in the total thyroidectomy group. The values of FNA-Tg in patients on and off therapy were 57.8 ng/ml (27.2–319.5) and 282 ng/ml (4.4–500.0), respectively (*p* = 0.74).

**TABLE 1:** Baseline characteristic of lymph nodes.

		With thyroid tissue	Without thyroid tissue	*p*
Age (years)		42.6 (13.2)	43.5 (12.9)	0.59
Size of LNs (cm)		1.2 (0.9–1.7)	1.3 (0.9–1.6)	0.57
FNA-Tg (ng/ml)		256.2 (1.1–500)	4.46 (0.2–23.2)	<0.001
FNAC Positive	Suspicious	10 (6)	2 (2)	0.085
	Malignant	252 (247)	40 (39)	
FNAC Negative	Benign	165 (29)	28 (7)	0.491
	Atypical	7 (3)	2 (2)	
	Inadequate	9 (4)	2 (1)	
Surgery	Positive	289	51	0.382
	Negative	154	23	

FNAC, fine needle aspiration cytology; FNA-Tg, thyroglobulin in the FNA, washout fluid. Data presented as mean (SD), median (IQR) or n (the number of cases of malignant confirmed by surgery).

**FIGURE1:**
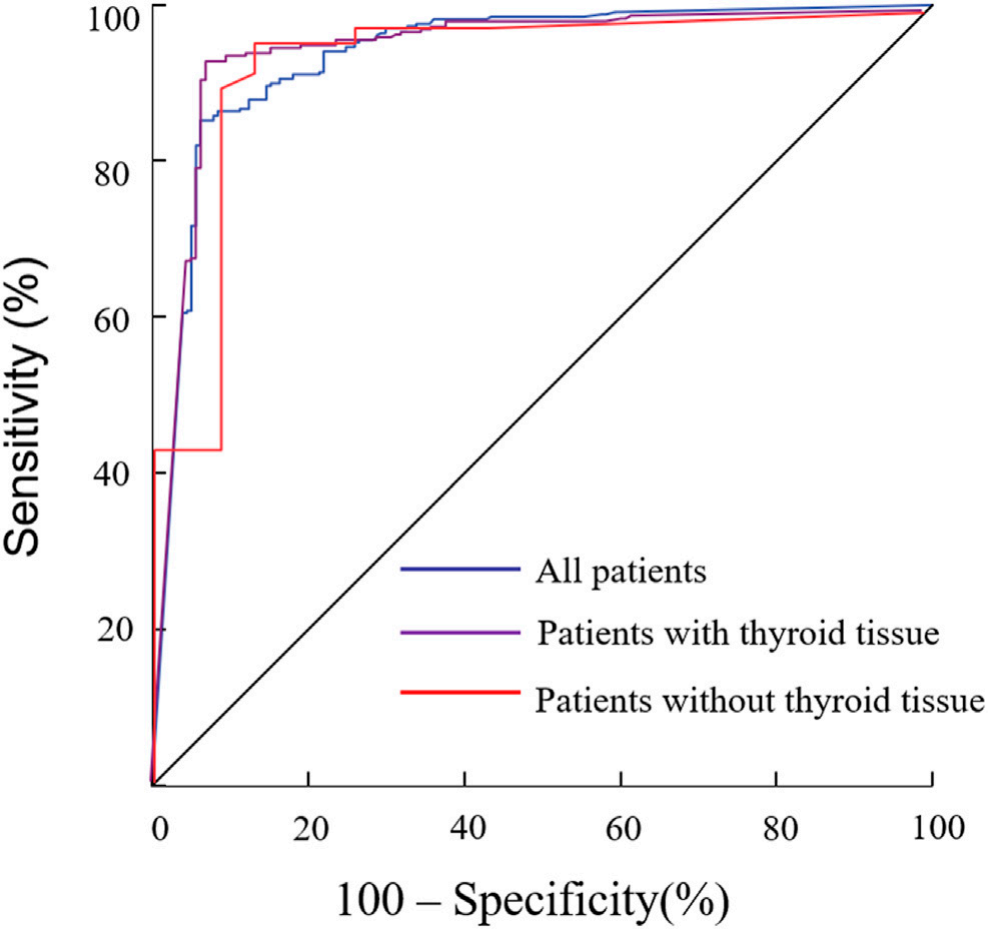
**(A)**, The ROC curve for all patients. The optimal cut-off value is 19.4 ng/ml and the AUC is 0.94 (95%, 0.91–0.96); **(B)**, The ROC curve for patients with thyroid tissue, and the optimal cut-off value is 19.4 ng/ml and the AUC is 0.95 (95%, 0.92–0.97); **(C)**, The ROC curve for patients without thyroid tissue, and the optimal cut-off value is 1.2 ng/ml and the AUC is 0.93 (95%, 0.85–0.98).

By constructing ROC curves, the optimal cut-off value for each group was determined according to the highest critical point of the Youden index (Figure 1). The optimal cut-off value of FNA-Tg in distinguishing benign LNs from malignant LNs was 19.4 ng/ml [sensitivity 85.3%; specificity 93.8%; AUC 0.94 (95% CI 0.91–0.96)] in all cases. The optimal cut-off value was higher in the group with thyroid tissue than those without thyroid tissue. In the group with thyroid tissue, the optimal cut-off value was still 19.4 ng/ml [sensitivity 93.4%, specificity 92.9%, AUC = 0.95 (0.92–0.97)]. In the group after total thyroidectomy, the optimal diagnostic threshold was 1.2 ng/ml [sensitivity 96.1%, specificity 87.0%; AUC = 0.93 (0.85–0.98)].

The commonly used FNA-Tg cut-off values and different values suggested in other studies were selected, and applied to this study according to different surgical conditions in order to compare the diagnostic performance. In the group with thyroid tissue, the 19.4 ng/ml was the optimal cut-off value (Table 2). However, the sensitivity of 1.0 ng/ml was highest, and the highest specificity was when FNA-Tg values were 28.5, 36, and 50 ng/ml. In the group without thyroid tissue, the highest sensitivity was 0.2 ng/ml, the highest specificity ≥19.4. The cut-off value with the best diagnostic accuracy was 1.2 ng/ml (Table 3).

**TABLE 2:** Comparison of diagnostic performance of various cut-off values of FNA-Tg with thyroid tissue.

FNA-Tg [n]	Sensitivity	Specificity	PPV	NPV	Accuracy
FNA-Tg [1.0]	97.2 (281/289)	64.9 (100/154)	83.9 (281/335)	92.6 (100/108)	86.0 (381/443)
FNA-Tg [2.2]	96.2 (278/289)	72.1 (111/154)	86.6 (278/321)	91.0 (111/122)	87.8 (389/443)
FNA-Tg [5.0]	95.5 (276/289)	79.9 (123/154)	89.9 (276/307)	90.4 (123/136)	90.1 (399/443)
FNA-Tg [10.0]	94.5 (273/289)	85.1 (131/154)	92.2 (273/296)	89.1 (131/147)	91.2 (404/443)
FNA-Tg [19.4]	93.4 (270/289)	92.9 (143/154)	96.1 (270/281)	88.3 (143/162)	93.2 (413/443)
FNA-Tg [28.5]	91.0 (263/289)	93.5 (144/154)	96.3 (263/273)	84.7 (144/170)	91.9 (407/443)
FNA-Tg [36.0]	90.3 (261/289)	93.5 (144/154)	96.3 (261/271)	83.7 (144/172)	91.4 (405/443)
FNA-Tg [50.0]	88.6 (256/289)	93.5 (144/154)	96.2 (256/266)	81.4 (144/177)	90.3 (400/443)
FNA-Tg > serum Tg	80.0 (196/245)	84.6 (55/65)	95.1 (196/206)	52.9 (55/104)	81.0 (251/310)

PPV: positive predictive value; NPV: negative predictive value; FNA-Tg, the value of thyroglobulin during fine needle aspiration biopsy washout fluid; FNA-Tg [1.0, 2.2, 5.0, 10.0, 19.4, 28.5, 36.0, 50.0], the cut-off value of interpretation for positivity test was 1.0, 2.2, 5.0, 10.0, 19.4, 28.5, 36.0, and 50.0 ng/ml; Serum Tg: the value of thyroglobulin in the serum. A surgery pathology result was used as the gold standard. Numbers in parentheses are the number of cases.

**TABLE 3:** Comparison of diagnostic performance of various cut-off values of FNA-Tg without thyroid tissue.

FNA-Tg [n]	Sensitivity	Specificity	PPV	NPV	Accuracy
FNA-Tg [0.2]	98.0 (50/51)	73.9 (17/23)	76.9 (50/56)	94.4 (17/18)	90.5 (67/74)
FNA-Tg [1.0]	96.1 (49/51)	82.6 (19/23)	92.5 (49/53)	90.5 (19/21)	91.9 (68/74)
FNA-Tg [1.2]	96.1 (49/51)	87.0 (20/23)	94.2 (49/52)	90.9 (20/22)	93.2 (69/74)
FNA-Tg [2.2]	86.3 (44/51)	91.3 (21/23)	95.7 (44/46)	75.0 (21/28)	87.8 (65/74)
FNA-Tg [5.0]	66.7 (34/51)	70.0 (16/23)	82.9 (34/41)	50.0 (16/32)	67.6 (50/74)
FNA-Tg [10.0]	51.0 (26/51)	70.0 (16/23)	78.8 (26/33)	39.0 (16/41)	56.8 (42/74)
FNA-Tg [19.4]	39.2 (20/51)	100 (23/23)	100 (20/20)	42.6 (23/54)	58.1 (43/74)
FNA-Tg [28.5]	29.4 (15/51)	100 (23/23)	100 (15/15)	39.0 (23/59)	51.4 (38/74)
FNA-Tg > serum Tg	90.9 (30/33)	87.0 (20/23)	90.9 (30/33)	87.0 (20/23)	89.2 (50/56)

PPV: positive predictive value; NPV: negative predictive value; FNA-Tg, the value of thyroglobulin in TNA, washout; FNA-Tg [0.2, 1.0, 1.2, 2.2, 5.0, 10.0, 19.4, 28.5], the cut-off value of interpretation for positivity test was 0.2, 1.0, 1.2, 2.2, 5.0, 10.0, 19.4, and 28.5 ng/ml; Serum Tg: the value of thyroglobulin in the serum. A surgery pathology result was used as the gold standard. Numbers in parentheses are the number of cases.

As the serum thyroglobulin level was not a routine preoperative measurement, the serum thyroglobulin value was measured in 310 cases in the thyroid tissue group (Table 2) and 56 cases in the thyroid tissue absence group (Table 3) 1–2 months before thyroid surgery. We analyzed the diagnostic performance of a FNA-Tg/thyroglobulin ratio >1. In the group with thyroid tissue, the sensitivity and specificity of the FNA-Tg/thyroglobulin ratio >1 were 80.4 and 84.6%, respectively, while in the non-thyroid tissue group, the sensitivity and specificity of the ratio were slightly higher at 90.9 and 87.0% respectively. This suggested that FNA-Tg/thyroglobulin ratio >1 has higher diagnostic value when the thyroid tissue was absent.

## Discussion

The measurement of FNA-Tg levels was first proposed by Pacini in 1992, and the mean ±2 times standard deviation of FNA-Tg of benign LN has been suggested to be taken as the best cut-off value for diagnosing with a sensitivity of up to 100% (13). Some have studied the cut-off values of FNA-Tg while most of the subjects in those have focused on post-operation data (15–18), whilst others analyzed influencing factors such as thyroglobulin, TSH and TgAb, but no consistent conclusions have been reached. Jeon et al. (17) have proposed that the cut-off value of FNA-Tg is different according to different thyroglobulin levels, while some studies showed that the serum thyroglobulin level had no effect on FNA-Tg (12, 19–21). Studies have showed no statistical difference in FNA-Tg values between TgAb positive group and the TgAb negative group (12, 20, 22–25). Some (17, 23) have proposed that the FNA-Tg value of metastatic LNs of thyroid cancer was lower or even undetectable if TgAb was positive. As we found no influence of serum thyroglobulin, TgAb and TSH on the value of FNA-Tg, we consider the status of thyroid tissue may be the main influencing factor compared with others.

There are few other studies that focused on the influence of the presence or absence of thyroid tissue on the cut-off value. Therefore, we mainly focus on the influence of status of the thyroid tissue. Non-parametric Kruskal-Wallis tests were used to confirm statistical difference in FNA-Tg values between the preoperative and the total thyroid tissue groups and between subtotal thyroidectomy and total thyroidectomy groups, while no statistical difference was observed in the FNA-Tg values between the preoperative and subtotal thyroidectomy groups. This indicated that the presence of thyroid tissue has an impact on FNA-Tg values, which was consistent with that of the conclusions put forwarded by Kim and Moon et al. (8, 14, 20, 26, 27). In this study, the optimal cut-off value was 19.4 ng/ml according to the FNA-Tg results of all patients. When thyroid tissue exists, the optimal cut-off value based on ROC curve was consistent with that of the optimal cut-off value without considering the thyroid status, and this was consistent with the study findings conducted by LEE et al. (16), in which the optimal threshold still remained to be 19.4 ng/ml, but with higher sensitivity and specificity irrespective of thyroid status. In the absence of thyroid tissue, the optimal cut-off value of FNA-Tg was 1.2 ng/ml. The optimal cut-off value of the thyroid tissue group was higher than that of non-thyroid tissue group, considering that the thyroglobulin secreted by thyroid follicular epithelial cells in normal thyroid tissue might enter into adjacent metastatic LNs via some other pathways.

Based on large scale recruitment of patients in our study, the cut-off values used in this study were those that are commonly used in the past literatures and are used for comparing the diagnostic performance. The commonly used values in the previous studies are FNA-Tg [1.0, 5.0, 10.0, 50.0] and FNA-Tg/thyroglobulin ratio >1. In several studies, 1.0 ng/ml is taken as the optimal cut-off value for diagnosing malignant LNs in PTC (15–17, 20) and some have suggested the use of FNA-Tg > thyroglobulin as the positive standard (16, 17, 20). According to the results of different studies, different cut-off values showed different diagnostic performances according to the thyroid gland status. Lee et al. (16) have suggested that 2.2 ng/ml and a FNA-Tg/thyroglobulin ratio >1 could be used as the cut-off value for the two groups, respectively. Others (12, 25) have proposed that 4.41 ng/ml and 28.5 ng/ml are the best diagnostic performance values regardless of the thyroid status. Some experts (20, 26,27) have suggested the use of 32, 36, and 39.3 ng/ml as positive standard values when thyroid tissue is present, and 0.9, 1.7, and 2.2 ng/ml as the optimal cut-off values in the absence of thyroid tissue. By analyzing and comparing, 1.0 ng/ml has shown the highest sensitivity (97.2%) in the group with thyroid tissue, consistent with the results obtained by Jeon et al. (17) with a sensitivity of 100%. The highest specificity was 28.5, 36.0, 50.0 ng/ml, both reaching up to 93.5%. The highest diagnostic sensitivity of thyroid tissue absence were 0.2, 1.0, and 1.2 ng/ml, the highest specificity were 19.4 and 28.5 ng/ml, and the best overall diagnostic efficacy was still 1.2 ng/ml. Considering the absence of thyroid tissue and incomplete thyroglobulin information, the FNA-Tg/thyroglobulin ratio >1 did not achieve optimal diagnostic efficacy. The preoperative cut-off values as suggested by other studies to the group with thyroid tissue were applied in this study, but 19.4 ng/ml still remained the cut-off value with the best diagnostic performance. The optimal cut-off value of FNA-Tg recommended by different studies was different, and this might be caused by the differences in study population and their respective diagnostic criteria. In addition, the diagnostic performances of FNAC, FNA-Tg alone, and their combination were compared. The diagnostic performance was significantly improved (specificity 94.4%, PPV 97.11%, NPV 95.5%, accuracy 96.5%) when used in combination than used alone (FNAC: sensitivity 86.5%, specificity 95.4%, PPV 97.4%, NPV 78.6%, accuracy 89.6%; FNA-Tg: sensitivity 93.8%, specificity 91.0%, PPV 95.2%, NPV 88.5%, accuracy 92.8%). Although FNA-Tg has good diagnostic performance, there is still a false positive rate (4.8%) and false negative rate (11.4%). At present, FNA-Tg cannot replace FNAC, and the combination of FNAC and FNA-Tg is more valuable, in line with Zhao et al. (8).

However, there are certain limitations in our study. First, there was a selection bias, because the patients who underwent FNAC and FNA-Tg had ultrasonic malignant signs. Secondly, there was a difference in the number of patients between the group with thyroid tissue and the group without thyroid tissue, and the number of cases collected in the two groups was not balanced in the same period. Therefore, it is necessary to further expand the sample number of patients after total thyroidectomy to reduce the interference of various uncertain factors.

Our data represent an advance in biomedical science in that it demonstrates a method for determining the likelihood of the malignant invasion of lymph nodes in thyroid cancer.

## Summary Table

### What is Known About This Subject

• Thyroglobulin measurement in the washout fluid of the needle used for FNAC aspiration (FNA-Tg) is a useful tool for improving the diagnostic accuracy of FNA.
• However, the optimal cut-off value of FNA-Tg is uncertain and the results of current studies are controversial.

### What This Paper Adds

• FNA-Tg of 19.4 ng/ml and 1.2 ng/ml can be used as optimal cut-off values for the presence or absence of thyroid tissue, respectively.
• FNA-Tg can used as an important auxiliary method for diagnosing cervical metastatic LNs of thyroid cancer.

## Data Availability

The original contributions presented in the study are included in the article/supplementary material, further inquiries can be directed to the corresponding author.
